# Identification of MALT1 as both a prognostic factor and a potential therapeutic target of regorafenib in cholangiocarcinoma patients

**DOI:** 10.18632/oncotarget.23049

**Published:** 2017-12-08

**Authors:** Chun-Nan Yeh, Yu-Chan Chang, Yeu Su, Dennis Shin-Shian Hsu, Chi-Tung Cheng, Ren-Chin Wu, Yi-Hsiu Chung, Kun-Chun Chiang, Ta-Sen Yeh, Meng-Lun Lu, Chun-Yu Liu, Peter Mu-Hsin Chang, Ming-Han Chen, Chi-Ying F. Huang, Michael Hsiao, Ming-Huang Chen

**Affiliations:** ^1^ Department of Surgery, Liver Research Center, Chang Gung Memorial Hospital, Chang Gung University, Taoyuan, Taiwan; ^2^ Genomics Research Center, Academia Sinica, Taipei, Taiwan; ^3^ Institute of Biopharmaceutical Sciences, National Yang-Ming University, Taipei, Taiwan; ^4^ Genome Research Center, National Yang-Ming University, Taipei, Taiwan; ^5^ Department of Pathology, Chang Gung Memorial Hospital, Chang Gung University, Taoyuan, Taiwan; ^6^ Center for Advanced Molecular Imaging and Translation, Chang Gung Memorial Hospital, Taoyuan, Taiwan; ^7^ Department of Oncology, Taipei Veterans General Hospital, Taipei, Taiwan; ^8^ School of Medicine, National Yang-Ming University, Taipei, Taiwan; ^9^ Department of Biochemistry, College of Medicine, Kaohsiung Medical University, Kaohsiung, Taiwan

**Keywords:** cholangiocarcinoma, regorafenib, MALT1, MI-2, Elk-1

## Abstract

Intrahepatic cholangiocarcinoma (CCA) is an aggressive cancer that lacks an effective targeted therapy. Here, we assessed the therapeutic efficacy of regorafenib in CCA, as well as elucidated its underlying mechanism. We first demonstrated that regorafenib not only inhibited growth but also induced apoptosis in human CCA cells. Subsequently, we used *in silico* approaches to identify MALT1 (Mucosa-associated lymphoid tissue protein 1), which plays an important role in activating NF-κB, as a potential target of regorafenib. Overexpression of *Elk-1,* but not *Ets-1,* in HuCCT1 cells markedly reduced their sensitivity to regorafenib, which might be attributed to a significant increase in MALT1 levels. Our results further demonstrated that this drug drastically inhibited MALT1 expression by suppressing the Raf/Erk/Elk-1 pathway. The efficacy of regorafenib in decreasing *in vivo* CCA growth was confirmed in animal models. Regorafenib efficacy was observed in two MALT1-positive CCA patients who failed to respond to several other lines of therapy. Finally, MALT1 was also identified as an independent poor prognostic factor for patients with intrahepatic CCA. In conclusion, our study identified MALT1 to be a downstream mediator of the Raf/Erk/Elk-1 pathway and suggested that MALT1 may be a new therapeutic target for successful treatment of CCA by regorafenib.

## INTRODUCTION

Intrahepatic cholangiocarcinoma (CCA) is the second most common primary hepatic tumor type, with increases in incidence and mortality worldwide in recent years [1–3]. Surgical resection is the only available curative therapy for patients with intrahepatic CCA. However, since most cases of CCA are diagnosed at advanced disease stages and/or when liver functions are already poor, palliative chemotherapy with gemcitabine and cisplatin is often the best treatment option, despite its limited effects [4]. While several molecular-targeted therapies have been evaluated in clinical trials, the results have been disappointing [5–7]. Collectively, no standard therapy has been set for refractory CCA, hence novel therapeutic drugs for its treatment are urgently needed.

Regorafenib is a potent oral inhibitor of multiple kinases which might be involved in tumor angiogenesis (VEGFR-1, -2, -3, Tie-2), oncogenesis (KIT, RET, RAF-1, BRAF, BRAFV600E), and tumor niche formation (PGDFR, FGFR). Regorafenib has recently been shown to be effective in treating several gastrointestinal (GI) tumors [8, 9]. Although CCA is not usually a hypervascular tumor, regorafenib is still considered to be a potential therapeutic agent against this disease since several molecular alterations, including the disruption of the MAPK pathway and the activation of Ras and BRAF mutations, have been described in CCA. In fact, several clinical trials are currently underway to investigate the efficacy of regorafenib in the treatment of CCA (NCT02162914, NCT02053376, and NCT02386397), although only marginal effects in CCA treatment have been reported when similar classes of multi-kinase inhibitors, such as sorafenib and sunitinib, were tested [10, 11].

The mucosa-associated lymphoid tissue protein 1 (MALT1) is an intracellular signaling protein that plays an essential role in the nuclear factor κB (NF-κB) pathway [12, 13] by functioning as a scaffold that helps assemble a complex that results in NF-κB activation [14]. In addition, MALT1 is also a paracaspase that can cleave some of its substrates such as A20, RelB, and NF-κB-inducing kinase (NIK) to enhance NF-κB activation [12, 15]. All these findings suggest that MALT1 is a promising therapeutic target for the treatment of cancers resulting from NF-κB activation. Relatedly, aberrant activation of NF-κB has recently been identified in intrahepatic CCA [16], but the involvement of MALT1 in this event is still unclear.

In this study, we investigated whether regorafenib suppresses *in vitro* and *in vivo* growth of CCA cells and dissected its mechanism of action. Our results initially showed that regorafenib inhibited the growth of HuCCT1 and KKU-100 human CCA cell and induced their apoptosis. We further found that the gene signatures of regorafenib-treated CCA cells were similar to those induced by MALT1 knockdown, suggesting that MALT1 may be a target of regorafenib. Our subsequent results indicated that regorafenib inhibited NF-κB activation by suppressing the Raf/Erk/Elk-1/MALT1 pathway. We also observed that two MALT1-positive patients received clinical benefits from regorafenib. Finally, we demonstrated, for the first time, that elevated MALT1 expression was a significant poor prognostic factor for patients with intrahepatic CCA. Taken together, our findings suggest that regorafenib might be useful in treating this malignancy by inhibiting MALT1-mediated NF-κB activation.

## RESULTS

### Regorafenib inhibits the growth of human CCA cells and induces their apoptosis

**Figure 1:**
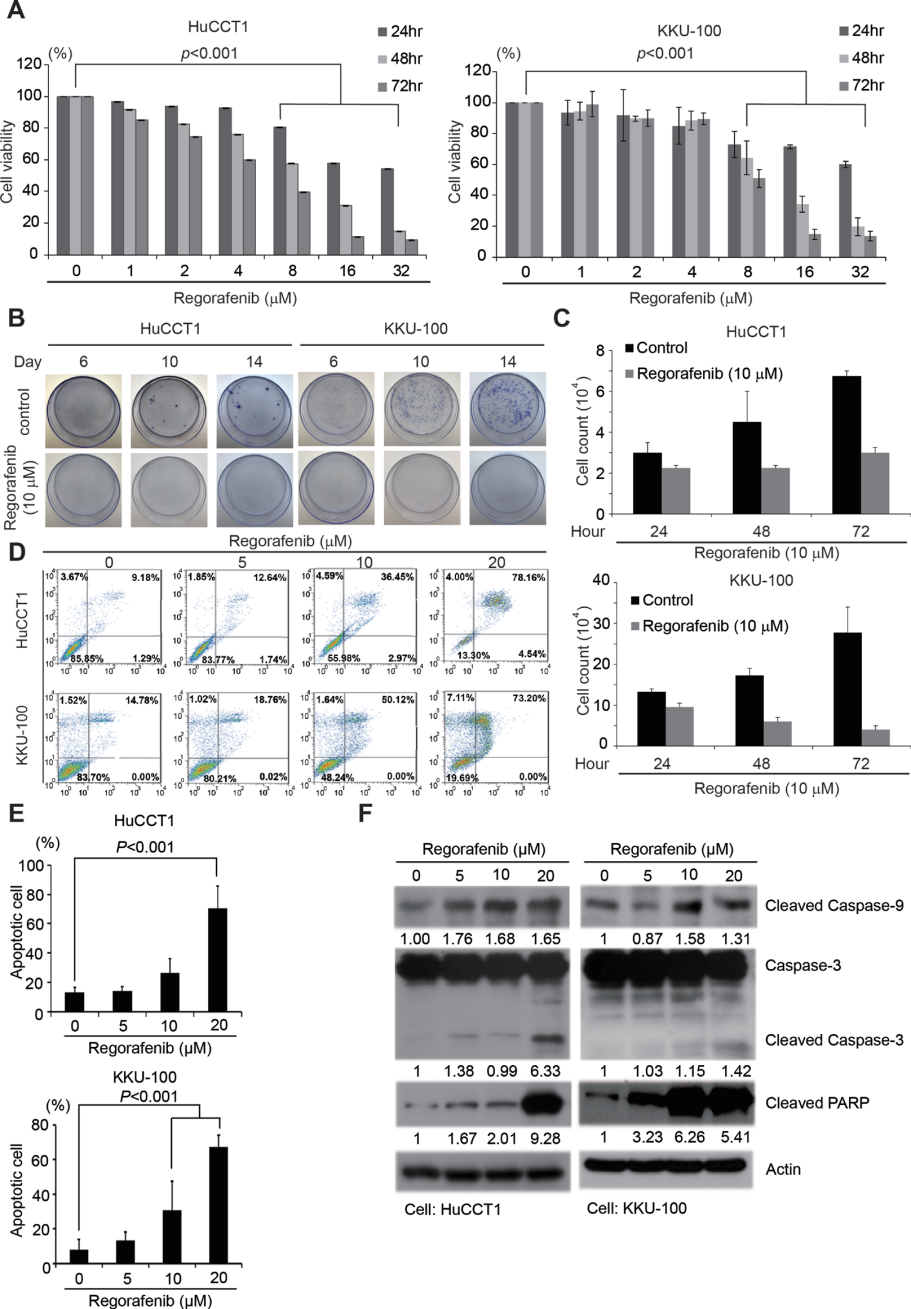
Regorafenib inhibited CCA cell growth and induced tumor cell apoptosis (**A**) HuCCT1 and KKU-100 cell lines were cultured with or without regorafenib at gradient concentrations for 24, 48 and 72 hrs. Cell viability was evaluated by MTT assay. Data represents the mean ± standard deviation of three independent experiments. (**B**) Colony formation assay in HuCCT1 and KKU-100 cells at 6, 10 and 14 days following treatment with or without 10 μM regorafenib. (**C**) Cell count assay in HuCCT1 and KKU-100 cells at 24, 48 and 72 hr by microscopy. (**D**) Quantitation of the propidium iodide (PI) percentage of HuccT1 and KKU-100 cells cultured with regorafenib at gradient concentration for 72 hrs through flow cytometry. (**E**) HuCCT1 and KKU-100 cells were treated with or without regorafenib at the indicated concentrations, 0, 5, 10 and 20 μM for 48 hrs. Apoptotic cells were measured using the TACS Annexin V-FITC apoptosis detection kit and are represented as a percentage of total events. (**F**) Western blot analysis of cleaved PARP, caspase 9, and caspase 3 in HuCCT1 and KKU-100 cells after treatment with or without regorafenib at the indicated concentrations 0, 5, 10 and 20 μM for 48 hrs. β-actin was used as an internal control for protein loading.

To determine the anti-proliferative effects of regorafenib in CCA cells, the growth of two human intrahepatic CCA cell lines, HuCCT1 and KKU-100, was analyzed by MTT assay and clonogenecity assay in the presence of varying concentrations of regorafenib. As shown in Figure 1A, regorafenib exhibited a concentration and time-dependent anti-proliferative effect in both HuCCT1 and KKU-100 cells, with IC_50_ values of 5.9 and 8.2 µM, respectively. The anti-proliferative effect of regorafenib was confirmed by clonogenecity assay (Figure 1B). We also confirmed that regorafenib had therapeutic efficiency by observing cell death in cholangiocarcinoma cells (Figure 1C). To confirm the apoptosis-inducing effect of regorafenib in human CCA cells, after treatment with varying concentrations of this drug, the percentages of apoptotic populations in HuCCT1 and KKU-100 cells were determined by FITC-Annexin V staining and subsequent flow cytometry. We observed that regorafenib treatment resulted in a concentration-dependent increase in apoptotic populations (Figure 1E). In fact, as many as 78.1% of HuCCT1 and 73.2% of KKU100 cells underwent apoptosis after being treated with 20 µM of regorafenib for 48 hrs (Figure 1D and 1E). Furthermore, 4% of HuCCT1 and 7.1% of KKU100 cells also underwent necrosis after being treated with 20 µM of regorafenib for 48 hrs (Figure 1D). The above speculation was further confirmed by the dose-dependent increase in cleaved forms of Caspase-3 and Caspase-9 as well as PARP in both cells (Figure 1F).

### MALT1 is a potential drug target of regorafenib and the growth of human CCA cells is also suppressed by the MALT1 inhibitor MI-2

To identify potential targets of regorafenib, we obtained the gene signatures of 3 CCA cell lines, HuCCT1, SNU-1079, and SNU-1196 after treatment with 10 µM of regorafenib for 6 hrs, using L1000 profiling database. *ECH1*, *MALT1*, and *ALAS1* were the top 3 perturbagen gene candidates by analysis of the gene signatures in LINCS dataset since their expression was affected by regorafenib treatment in all three CCA cell lines (Supplementary Table 1). The basic finding from LINCS is that gene expression from regorafenib is similar to gene expression from knockdown MALT1, ECH1 or ALAS1. Therefore, we searched for the gene which decreased after regorafenib treatment.

**Figure 2:**
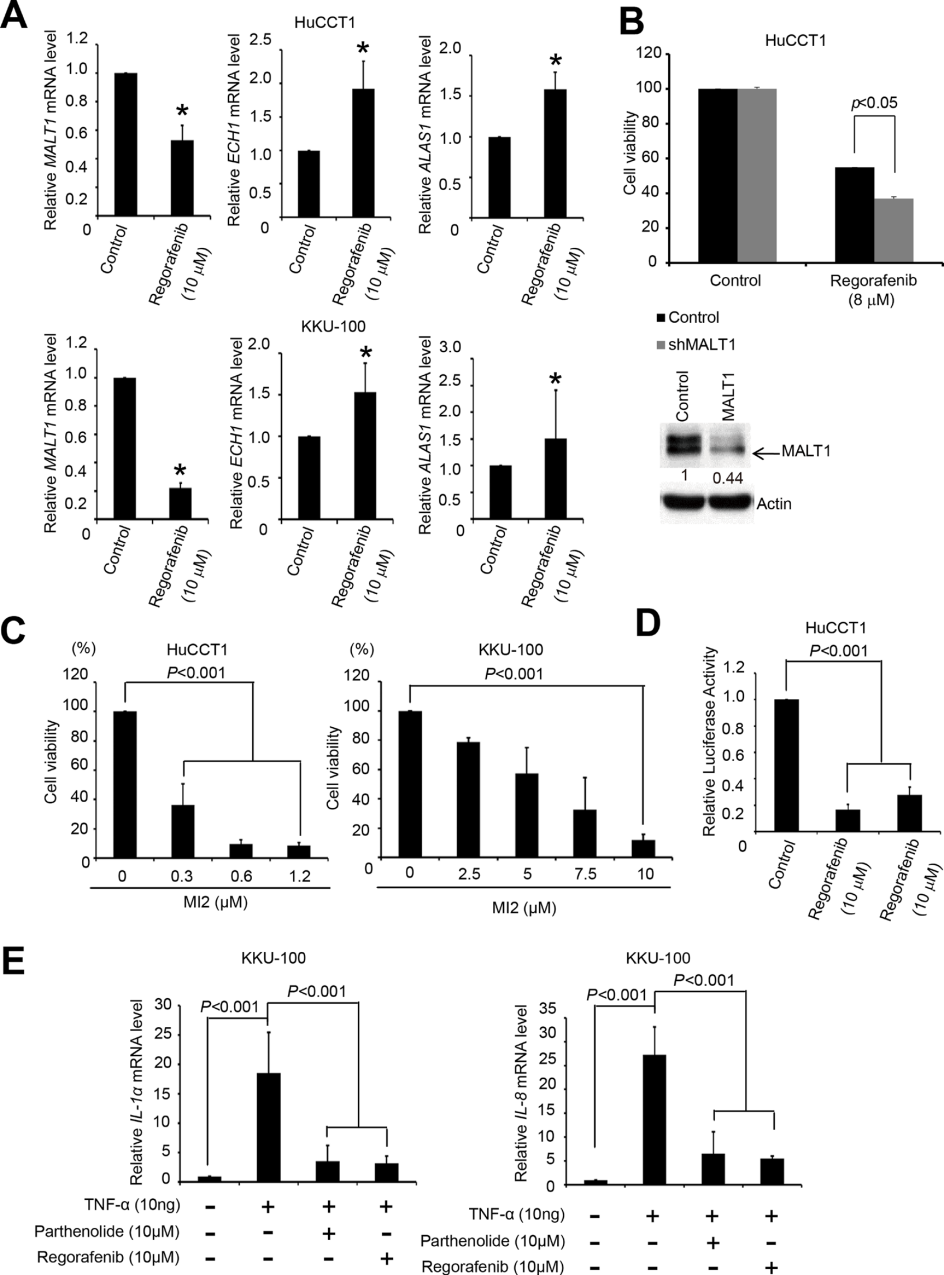
Regorafenib inhibited MALT1 expression and NF-κB pathway (**A**) HuCCT1 and KKU100 cells were cultured with or without regorafenib (10 μM) for 48 h and lysed with TRI reagent. Total RNA was harvested for reverse transcription. The mRNA levels of *ECH1*, *MALT1*, *ALAS1,* and *GAPDH* were then analyzed by quantitative PCR. All data were normalized to *GAPDH* (internal control). (**B**) Upper panels shows drug sensitivities of the LacZ- and *MALT1*-knockdown HuCCT1 cells were analyzed by MTT-based viability assays 72 h after treatment 8 μM regorafenib. Lower panel shows Immunoblot analyse of MALT1. β-actin is the internal protein loading control. (**C**) HuCCT1 and KKU100 cell lines were cultured with or without MI-2 at the indicated concentrations for 72 h. Cell viability was evaluated by MTT assay. (**D**) HuCCT1 cells were cultured with or without 10 μM parthenolide, and 10 μM regorafenib for 24 h; NF-κB activity was evaluated with transient luciferase assays. (**E**) KKU100 cells were cultured with or without 10 μM TNF-a, 10 μM TNF-a plus parthenolide, and 10 μM TNF-a plus 10 μM regorafenib for 16 h. The NF-κB target genes *IL1a* and *IL8* were measured by RT-qPCR. Data represent the mean ± standard deviation of three independent experiments (^*^P < 0.05 analyzed by student’s *t*-test.)

To determine which gene was the most likely target of regulation by regorafenib, qRT-PCR was performed to measure the mRNA levels of *ECH1, MALT1*, and *ALAS1* in both HuCCT1 and KKU-100 cells, before and after regorafenib treatment. As shown in Figure 2A, significant decreases in *MALT1* mRNA levels were detected in these cells after regorafenib treatment, whereas the expression levels of *ECH1* and *ALAS1* were increased and unaffected by this drug in HuCCT1 and KKU-100 cells, respectively. Hence, we hypothesized that *MALT1* is a likely target of regorafenib. To confirm that MALT1 is indeed a drug target in human CCA cells, *MALT1*-knockdown HuCCT1 cells were more sensitive to regorafenib when compared with cells transfected with an empty vector (Figure 2B). The cytotoxic effects of a MALT1 inhibitor, MI-2, which irreversibly suppresses the protease activity of MALT1 by direct binding, were measured by MTT assays in both HuCCT1 and KKU-100 cellsy binding to it on both HuCCT1 and KKU-100 cells were measured by MTT assays [17]. As shown in Figure 2C, MI-2 exhibited dose-dependent cytotoxic effects in HuCCT1 and KKU100 cells, with IC_50_ values of 0.24 and 5.76 μM, respectively.

### Regorafenib inhibits NF-κB activity in human CCA cells

Since the major role of MALT1 in cells is to activate the NF-κB pathway, and its expression was inhibited by regorafenib, we next asked whether regorafenib inhibited NF-κB activation in human CCA cells. We analyzed luciferase activities in HuCCT1 cells after transient transfection with a reporter gene controlled by NF-κB. Both regorafenib and the NF-κB inhibitor, parthenolide, significantly decreased luciferase activity (Figure 2D). We further examined the effects of regorafenib and parthenolide on the expression of two well-known NF-κB target genes, *IL-1α* and *IL-8*, in KKU-100 cells treated with recombinant TNF-α [18, 19]. As shown in Figure 2E, *IL-1α* and *IL-8* mRNA levels induction by TNF-α were markedly suppressed by these two drugs. Collectively, these results support the notion that regorafenib effectively inhibits NF-κB activity in human CCA cells.

### Regorafenib inhibits *MALT1* expression via suppressing the Raf-1/Erk/Elk-1 pathway

**Figure 3:**
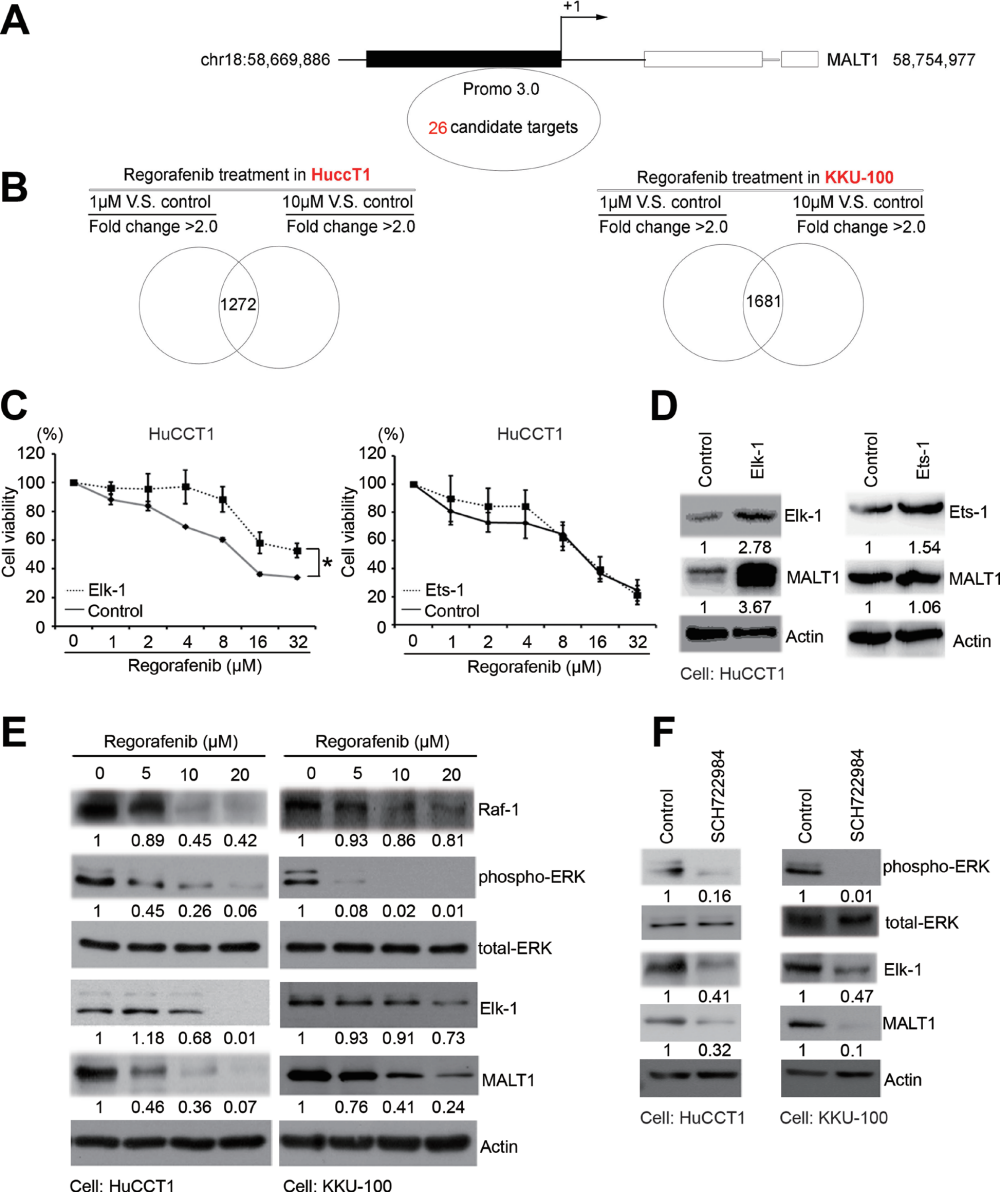
Regorafenib inhibited MALT1 through the RAF/ERK/Elk-1 pathway (**A**) The 26 putative *MALT1* promoter regions from roughly –1500 to +660 base pairs relative to the transcription start site were analyzed by PROMO 3.0 software. (**B**) The gene signatures of HuCCT1 and KKU-100 cells cultured without or with low doses (1 μM) or high doses (10 μM) of regorafenib for 6 h were analyzed by *Ingenuity Pathway Analysis* (IPA). (**C**) Drug sensitivities of the LacZ- and Elk-1 or ETS1-overexpressing cells were analyzed by MTT-based viability assays 72 h after treatment with the indicated concentrations of regorafenib. (**D**) Immunoblot analyses of Elk-1, ETS1, MALT1, with tubulin loading controls. (**E**) Immunoblot analyses of RAF-1, p-ERK, ERK, Elk-1, and MALT1, with or without regorafenib dose-dependent treatment in HuCCT1 and KKU-100 cells. β-actin is the internal protein loading control. (**F**) Immunoblot analyses of p-ERK, ERK, Elk-1, and MALT1 with or without SCH722984 treatment in HuCCT1 and KKU-100 cells. β-actin is the internal protein loading control (^*^P < 0.05 analyzed by student’s *t*-test.)

To dissect the molecular mechanisms underlying regorafenib-mediated inhibition of *MALT1* expression, potential transcription factor-binding sites on the *MALT1* promoter region (–1500 to +660 base pairs relative to the transcription start site) were searched with PROMO 3.0 software (http://alggen.lsi.upc.es/cgi-bin/promo_v3/promo/promoinit.cgi?dirDB=TF_8.3) and 26 such sites were identified (Figure 3A and Supplementary Table 2) [20, 21]. In addition, IPA software was utilized to find the common genes inhibited by regorafenib treatment in both HuCCT1 and KKU-100 cells (Figure 3B). After a combined analyses of the above results, we found that only ETS Proto-Oncogene 1 *(ETS-1)* and *ELK-1* fulfilled the criteria of Z score <–1 in two CCA cell lines, suggesting that ETS-1 and ELK-1 were the most likely transcription factors activating *MALT1* expression in these cells (Table 1). To determine the respective roles of ETS1 and ELK-1 in regulating *MALT1* expression in human CCA cells, MALT1 protein levels in HuCCT1 cells were examined after they were transiently transfected with expression vectors carrying the *ETS-1* and *ELK-1* genes. As shown in Figure 3D, a significant increase of MALT1 protein level was only detected in cells transfected with the *ELK-1*-expressing vector. In accordance with this, *ELK-1*-overexpressing HuCCT1 cells were more resistant to regorafenib when compared with cells transfected with an empty vector, whereas overexpression of *ETS-1* failed to affect their responses to this drug (Figure 3C). Since ELK-1 is a well-known downstream effector of the Raf/Erk pathway [22], we next asked whether regorafenib inhibited *MALT1* expression in human CCA cells via suppression of this pathway. As shown in Figure 3E, protein levels of Raf-1, phospho-Erk, Elk-1, and MALT1 in both HuCCT1 and KKU-100 cells were decreased in a dose-dependent manner by regorafenib. Furthermore, as shown in Figure 3F, protein levels of phospho-Erk, Elk-1 and MALT1 were also inhibited by the ERK inhibitor SCH772984. These results strongly suggested that the inhibition of *MALT1* expression in human CCA cells by regorafenib was through its suppression of the Raf-1/Erk/Elk-1 pathway.

**Table 1:** Predicted transcription factor binding sites of *MALT1* by PROMO 3.0 and Ingenuity Pathway Analysis (IPA)

	Regorafenib treatment in HuCCT1			Regorafenib treatment in KKU100		
	1 μM vs. control	10 μM vs. Control		1 μM vs. control	10 μM vs. control	
Upstream Regulator	*Z*-score	*Z*-score	*p*-value	*Z*-score	*Z-*score	*p*-value
XBP1	1.458	2.439	1.26E–05	–2.217	–0.636	1.24E–02
YY1	–0.152	–0.152	4.61E–05	–0.093	–0.093	8.36E–05
NR3C1	–1.757	–1.757	9.65E–21	–0.244	–1.672	1.06E–15
ESR1	–2.437	–2.923	6.98E–15	1.689	–0.095	8.04E–15
SP1	0.918	0.131	7.49E–10	–0.738	0.724	1.95E–06
**ELK1**	**–1.944**	**–1.285**	**1.15E–04**	**–2.731**	**–2.145**	**2.75E–04**
STAT1	3.073	3.447	3.97E–13	–0.160	1.269	8.51E–03
CEBPA	0.294	0.752	2.20E–07	–2.046	–0.409	1.71E–09
**ETS1**	**–1.634**	**–1.634**	**5.41E–06**	**–2.713**	**–2.274**	**2.69E–07**
JUN	–0.924	–0.089	2.06E–13	–1.228	0.577	1.51E–02
Ap1	–1.684	–0.621	2.80E–09	–2.736	–1.008	4.12E–11
STAT4	–0.064	0.959	7.55E–04	–0.246	0.678	1.01E–03
CEBPB	0.343	0.146	2.66E–08	–0.084	1.022	9.33E–05

### Regorafenib inhibits the *in vivo* growth of CCA cells in two animal models

**Figure 4:**
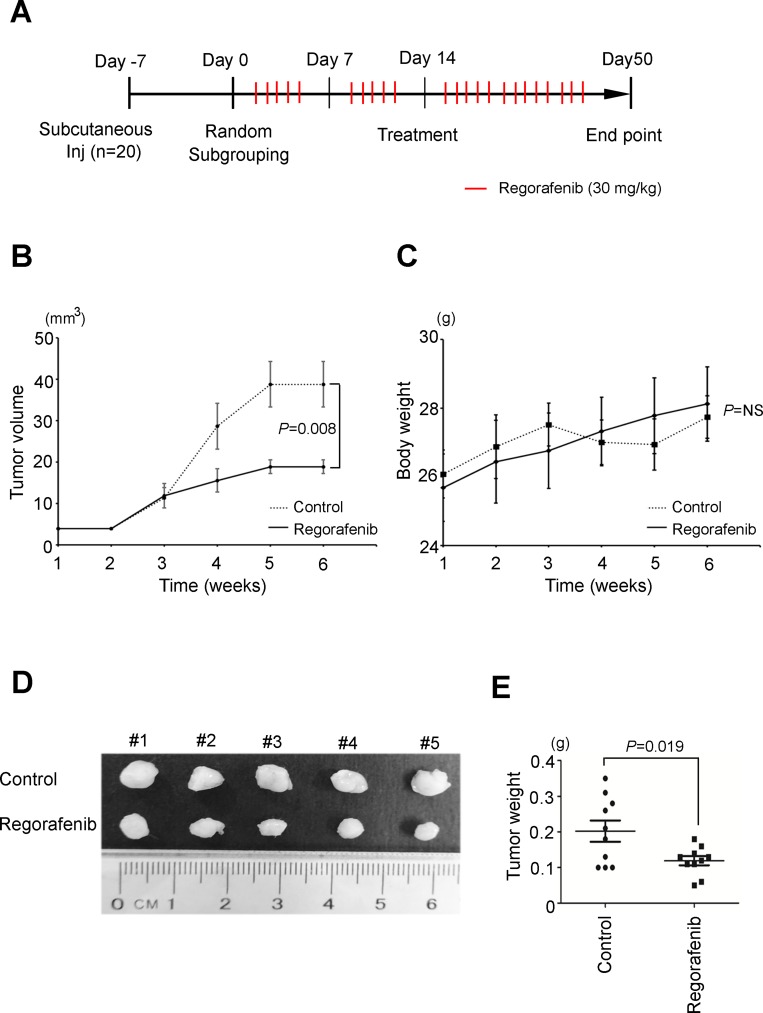
Regorafenib suppresses cholangiocarcinoma tumorigenicity *in vivo* (**A**) The flow chart of xenograft animal model treated with or without regorafenib. (regorafenib dose 30 mg/kg, 5 times per week, oral bleeding). (**B**) Overview of the solid tumor growth between regorafenib (30 mg/kg, dotted line) treatments compared with the control group (PBS, solid line) *in vivo*. (*P* = 8 × 10^3^, *n =* 5). (**C**) The body weights between regorafenib and control groups were similar. (**D**) Representative photographs of tumor size (*n =* 5 per treatment group). (**E**) Significantly lower tumor weight was found in the regorafenib group compared to the control group (*P* = 0.019). The statistical significances in control and regorafenib groups were analyzed by paired *t*-test.

To evaluate the effectiveness and safety of regorafenib treatment *in vivo*, we treated mice with regorafenib (30 mg/kg, five times a week by oral tube feeding) one week after they were xenografted with 1 × 10^6^ SNU-308 human CCA cells (Figure 4A). Tumor volume and body weight were monitored on a weekly basis. We observed that regorafenib treatment significantly suppressed the growth of SNU-308 cells *in vivo* compared with the vehicle group (Figure 4B, 4D and 4E). Moreover, no apparent changes in the body weight were detected in either group of mice during the treatment period, indicating that the dosage of regorafenib applied was not toxic to these animals (Figure 4C). We next used thioacetamide (TAA)-induced CCA in rats, as another animal model to assess the *in vivo* therapeutic efficacy of regorafenib, as well as a combination of gemcitabine and oxaliplatin (a standard therapy for CCA) on this malignancy. As shown in Supplementary Figure 1, each group presented with at least one FDG-avid tumor in the liver after 20 weeks of TAA treatment, based on visualization animal PET-CT in the coronal view. Rats were given vehicle, regorafenib alone, and gemcitabine plus oxaliplatin for 4 weeks. The tumor-to-liver (T/L) ratio of the SUV showed a steady elevation in the vehicle group after treatment (10.5 to 20.0% from 2 to 4 weeks). By contrast, significant decreases in the T/L ratio of the SUV elevation were clearly observed after two to four weeks of regorafenib and gemcitabine/oxaliplatin treatments (*P* < 0.05). Taken together, these results demonstrate that regorafenib treatment significantly suppressed the *in vivo* growth of CCA tumors in two animal models.

### Survival and prognostic analysis of CCA patients

**Figure 5:**
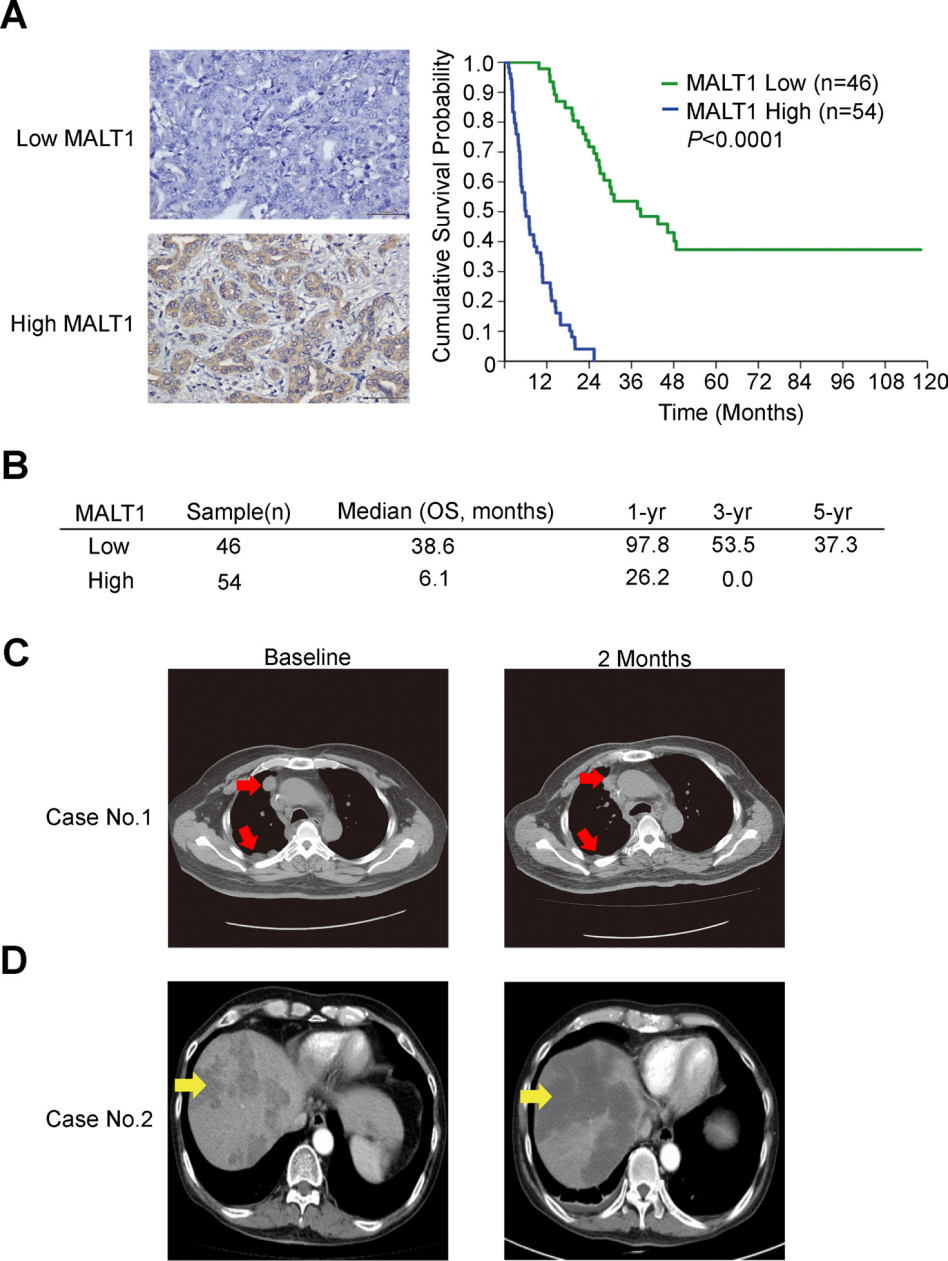
MALT1 expression correlated with worse survival in patients with resectable CCA (**A**) Upper-left and lower-left panels show representative low and high MALT1 immunohistochemical staining intensities respectively. Scale bar = 50 µm. Panel on the right shows the Kaplan–Meier plot of overall survival (OS) in patients with resectable MF-CCA tumors, based on their MALT1 expression levels. (**B**) The median OS, 1-year, 3-year, and 5-year survival rates in patients with resectable MF-CCA tumors, based on their MALT1 expression levels. (**C**) A 58-year-old man with metastatic CCA with a partial remission after regorafenib treatment. Before the start of treatment, computed tomographic (CT) images without intravenous contrast demonstrated pleural seeding and lymph node metastasis (white arrows). Follow-up CT images obtained 2 months after the start of therapy demonstrated regression of these lesions (white arrows). (**D**) A 50-year-old man with metastatic CCA with a stable disease after regorafenib treatment. Before the start of treatment, computed tomographic (CT) images with intravenous contrast demonstrated multiple liver metastases (yellow arrows). Follow-up CT images obtained 2 months after the start of therapy demonstrated marked tumor necrosis of these lesions (yellow arrows).

Fifty-four out of 100 mass-forming type CCA (MF-CCA) patient specimens (54%) revealed high cytoplasmic immunostaining for MALT1 (H score ≥120, Figure 5A). Interestingly, overexpression of MALT1 was found to be associated with symptoms, elevated alkaline phosphatase, and positive resection margin (Supplementary Table 3). Moreover, univariate log-rank analysis of 100 post-hepatectomy patients with MF-CCA identified the following factors as adverse influences on OS (overall survival): the presence of symptoms, elevated alkaline phosphatase, decreased albumin, tumor size >5 cm, positive surgical margin and lymph node status, and MALT1 immunostaining (Supplementary Table 4). However, multivariate Cox proportional hazard analysis demonstrated that non-curative hepatectomy and positive MALT1 immunostaining both independently predicted an inferior OS rate for MF-CCA patients after hepatectomy (Supplementary Table 5, Figure 5A and 5B).

### Preliminary clinical observation of the anti-CCA activity of regorafenib

One patient with metastatic CCA experienced a partial radiographic response to regorafenib that lasted for 5 months (Figure 5C). Another CCA patient showed a stable disease for 3.8 months, as well as tumor necrosis after regorafenib treatment (Figure 5D). Interestingly, the tumor tissues of both cases were MALT1-positive (Supplementary Figure 2).

**Figure 6:**
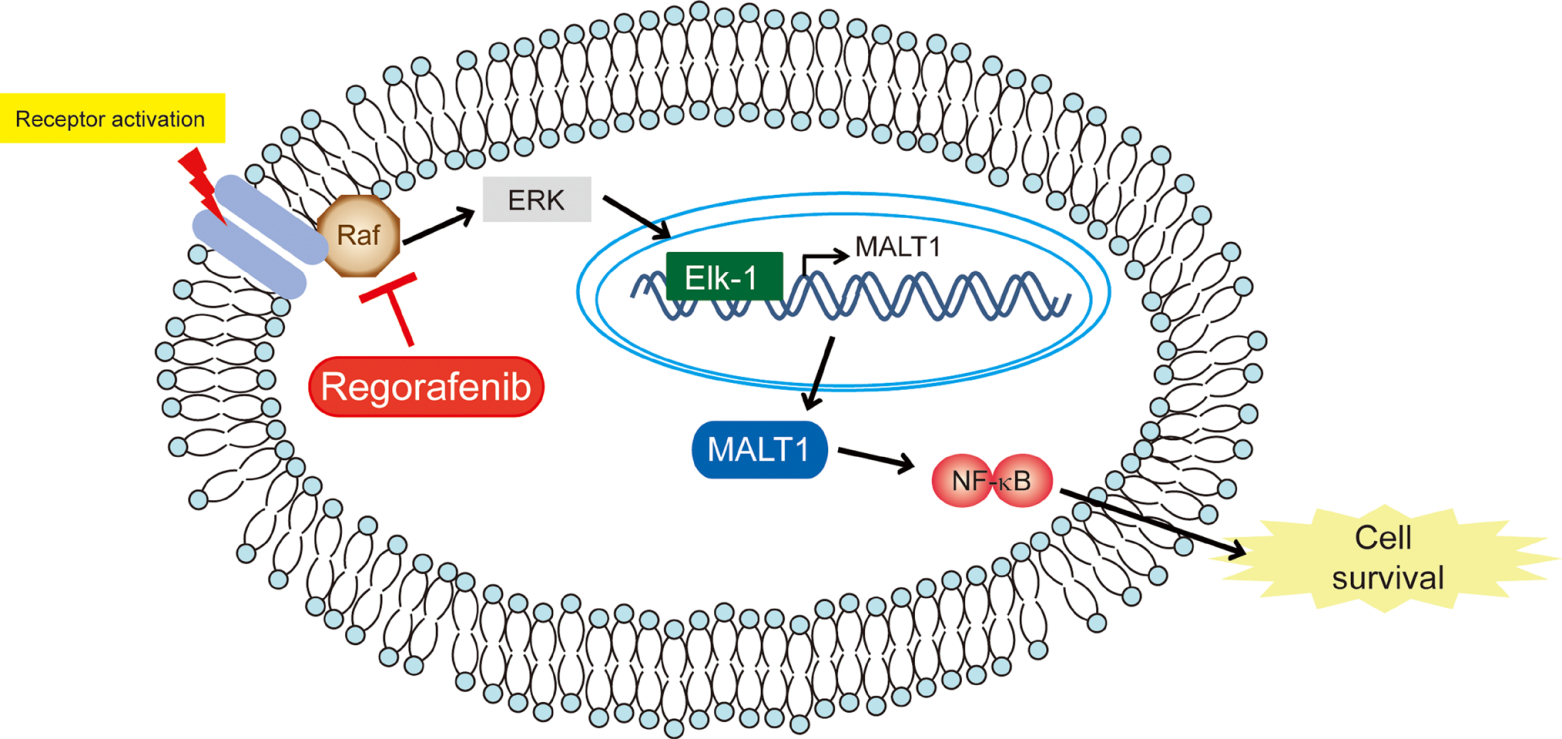
Schematic representation of the effects of regorafenib on CCA Schematic representation of the effects of regorafenib on CCA.

Based on this combined evidence, we concluded that regorafenib inhibited CCA growth in both *in vitro* and *in vivo* experiments, which is very likely via downregulation of MALT1 expression through suppression of the Raf/Erk/Elk-1 pathway. Most importantly, we demonstrated for the first time that MALT1 is a poor prognostic factor for patients with intrahepatic CCA who underwent hepatectomies. Collectively, our results suggest that MALT1 may be a new therapeutic target in CCA and that regorafenib is a potentially effective drug for this deadly malignancy by diminishing MALT1 expression (Figure 6).

## DISCUSSION

In this study, we demonstrated that regorafenib not only inhibited the proliferation but also induced the apoptosis of two human CCA cell lines. Moreover, we confirmed the *in vivo* efficacy of regorafenib in CCA therapy in two different animal models — a xenograft mouse model and a TAA-induced CCA rat model [23, 24]. Interestingly, MALT1 was found by the Library of Integrated Cellular Signatures (LINCS), an *in silico* method, to be a potential target of regorafenib in human CCA cells.

Regorafenib is a multi-targeted tyrosine kinase inhibitor of VEGFR-1/2/3, KIT, RET, RAF-1, BRAF, PGDFR, and FGFR. CCA is not usually a hypervascular tumor, but regorafenib is still considered a potential therapeutic agent for this disease since several molecular alterations, including the disruption of the MAPK pathway and the activation of Ras and BRAF mutations, have been described in CCA [25–27]. However, another similar multi-targeted tyrosine kinase inhibitor with multiple targets, sorafenib, has been shown to have a limited effect in CCA [10]. Therefore, there might be another important drug target in CCA, such as MALT1.

MALT1 plays two roles in the activation of NF-κB pathway [16]. One is to serve as a scaffold in the so-called CBM complex formed between CARMA1 (or CARM3 or CAR9), Bcl-10 and MALT1 which eventually results in the degradation of IκB and nuclear translocation of NF-κB [28]. In addition, MALT1 is a paracaspase which can cleave three NF-κB inhibitors, A20 [29], CYLD [30] and RelB [28] , to activate NF-κB. To no surprise, MALT1 hyperactivation has been associated with several types of cancers, including MALT lymphoma [31] and the activated B-cell (ABC) subtype of diffuse large B-cell lymphoma (DLBCL) [32–34]. MALT1 has recently been proposed to be a promising therapeutic target in autoimmunity and B cell lymphomas [35] and a current approach is to target the proteolytic functions of MALT1. However, the first inhibitor tested, Z-VRPR-FMK, is not suitable for clinical use due to its low potency and poor cell permeability [36]. Similarly, the phenothiazines, although able to selectively inhibit the cleavage activity of MALT1, are of limited clinical use due to their CNS side effects [37]. MI-2 which was shown to bind directly to MALT1 and acted as an irreversible inhibitor, could suppress the growth of activated B cell-like diffuse large B cell lymphoma (ABC-DLBCL) in both *in vitro* and *in vivo* assays [17]. To confirm that MALT1 is also a therapeutic target of CCA, we showed that *MALT1*-knockdown HuCCT1 cells are more sensitive to regorafenib (Figure 2B) and MI-2 inhibits the proliferation of CCA cell lines (Figure 2C).

In this study, we used a bioinformatics approach to find that regorafenib inhibits MALT1 protein production by suppressing the Raf/Erk/Elk-1 pathway, which strongly suggests that regorafenib can inhibit both the scaffold and proteolytic functions of MALT1. Furthermore, since regorafenib has already been approved by the U.S. FDA for the treatments of colon cancer and gastrointestinal stromal tumor [8, 9] utilization of this drug in treating MALT1-related cancers such as ABC-DLBCL should be easily adopted.

CCA cell apoptosis was induced by regorafenib through the caspase-mediated mitochondrial pathway, as demonstrated by elevated levels of cleaved caspase 3, 9 and PARP (Figure 1F). This result suggests that regorafenib inhibition of tumor cell death might result from apoptosis. Furthermore, 4% of HuCCT1 and 7.1% of KKU100 cells also underwent necrosis after treatment with 20 µM of regorafenib for 48 hrs (Figure 1D). Two patients who were refractory to several earlier lines of chemotherapy still benefited from regorafenib treatment (Figure 5C and 5D). More strikingly, central necrosis of the tumor was observed in one patient, which although very common in GIST patients treated with tyrosine kinase inhibitors, is very rare in CCA (Figure 5D). This finding was very similar to Huynh’s finding in patient-derived xenograft models of gastric cancer [38]. Since tumor tissues from both patients were MALT1-positive, this implies that MALT1 may have the potential to be a predictive biomarker for response to regorafenib (Supplementary Figure 2). In conclusion, regorafenib was effective in CCA, with inhibition of tumor growth and tumor-cell proliferation observed in *in vitro* and *in vivo* studies. MALT1 may be a downstream mediator of the Raf/Erk/Elk-1 pathway and therefore could be a new therapeutic target for successful treatment of CCA by regorafenib. These findings support further clinical investigation of regorafenib in CCA.

## MATERIALS AND METHODS

### Cell culture and reagents

The CCA cell lines HuCCT1 and KKU-100 were obtained from Japanese Collection of Research Bioresources Cell Bank (JCRB; Osaka, Japan). Another CCA cell line SNU-308 was obtained from Korean Cell Line Bank (Seoul, Korea). HuCCT1 and SNU-308 were cultured in RPMI1640 medium (Gibco/Thermo, CA, USA), and KKU-100 cells were cultured in Dulbecco-modified Eagle medium (DMEM; Gibco/Thermo, CA, USA). All cells were supplemented with 10% heat-inactivated fetal bovine serum, 100 μg/mL streptomycin, 100 μg/mL penicillin, and 2 mM L-glutamine in a humidified atmosphere containing 5% CO_2_ at 37°C. The anticancer agents and chemicals used include regorafenib, MI-2 and SCH722984 were commercially purchased (Selleck Chemicals, Houston, TX).

### Cell viability measurements

Cell viability was determined using the TACS tetrazolium salt 3-(4,5-dimethylthiazol-2-yl)-2, 5-diphenyltetrazolium bromide (MTT) cell proliferation assay kit (Trevigen, Gaithersburg, MD, USA) according to manufacturer’s instructions. MTT is used to determine cell viability in cell proliferation and cytotoxicity assays. The cells were seeded at a concentration of 2,000 cells/100 μL culture media per well into 96-well microplates. At 24 hours post-seeding, the cells were treated with dimethyl sulfoxide (DMSO) solvent control or different doses of regorafenib for 24, 48, or 72 hours. Subsequently, the cells were incubated in medium containing MTT for 4 hours, lysed by DMSO, and the optical density at 570 nm was measured using a microplate reader (Spectral Max250; Molecular Devices, Sunnyvale, CA, USA).

### Cell count proliferation assay

1 × 10^4^ cells of HuCCT1 and KKU-100 were seeded into 6 cm plates separately. Cells were treated by Regorafenib 10 μM for 24, 48, 72 hours. At each time point, cells were harvested, stained by trypan blue to exclude dead cells, The cells were calculated by Hemacytometer (Hausser Scientific).

### Clonogenecity assay

1,000 cells of HuCCT1 and 400 cells of KKU-100 were seeded into 6 cm plates separately. After treatment with Regorafenib 10 μM for 6, 10, and 14 days, cells were washed by cold PBS twice and fixed by 4% paraformaldehyde for 1 hour. Cells then were stained by 1% crystal violet for 1 hour. Purple colonies of cells were captured.

### Quantification of apoptotic cell death

Cells were plated on 6-well plates at a density of 7,000 per well and allowed to adhere overnight. After cell adhesion, cells were treated with DMSO or regorafenib at different doses for 48 hours. For flow cytometry, cells were trypsinized, harvested, fixed in 70% ethanol at –20°C, washed, and incubated with 10 mg/mL RNase A (Sigma Aldrich, St. Louis, MO, USA) for 15 minutes at 37°C. Next, the cells were stained with 200 μg/mL propidium iodide (Sigma Aldrich) for 1 hour at room temperature. Cells were evaluated using a FACS Calibur system (Becton Dickinson, Franklin Lakes, NJ, USA), and the data were analyzed by using CellQuest software (Becton Dickinson) to determine cell cycle distribution percentage. All experiments were performed in triplicates, and the data were expressed as the mean ± standard deviation.

### L1000 and LINCS analysis

L1000 is an innovative gene expression profiling technique with high-throughput scale (20 × 384 samples per week) for next-generation pharmaceutical discovery applications. Using L1000 profiling, disease indications can be linked with potential lead compounds, and genetic perturbagens can be generated using dedicated pattern-matching algorithms in the Library of Integrated Network-based Cellular Signatures (LINCS; http://www.broadinstitute.org/LINCS/dataset.html). The LINCS is an innovative gene expression profiling solution for next-generation pharmaceutical discovery applications and is a high-throughput (20 × 384 sample per week) low-cost (∼15% of regular array costs) gene expression profiling platform built at the Broad Institute [39, 40]. Using L1000 profiling, expression data generated from a large collection of small molecules is accessible through a Google-like search engine, allowing for disease indications to be connected with potential lead compounds by dedicated pattern-matching algorithms. The LINCS dataset includes 3,000 human genes, including known targets of FDA-approved drugs, drug-target pathway members, and candidate disease genes, which were perturbed using lentivirally delivered shRNAs in the same set of 15 cell lines. Supplementary Table 1 lists the gene perturbagen candidates, which were generated from a query of the regorafenib treatment gene signature in LINCS. The perturbagen candidates were then prioritized by their connectivity score across the four cell lines in which the perturbagen gene signature connected most strongly to that of regorafenib treatment and cut off at a connectivity score ≥ 90.

### Real-time reverse transcriptase PCR

Cellular total RNA was isolated using TRI reagent (Sigma Aldrich). 3 μg of total RNA was used to generate complementary DNA by SuperScript III reverse transcriptase (Thermo Fisher, Waltham, MA, USA), and Maxima SYBR Green qPCR Master Mix (Thermo Fisher, Waltham, MA, USA) was used to perform real-time PCR. The target mRNA and β-actin (as the internal control) were analyzed using a LightCycler 480 system (Roche, Basel, Switzerland). The primer sets of qPCR were listed in Supplementary Table 6. All experiments were performed in triplicates and repeated three times.

### Western blot analysis

Whole cell lysates from CCA cell lines were obtained by using Pierce immunoprecipitation assay buffer (Thermo Scientific, Rockford, IL, USA). Protein samples were separated on 8%–12% gradient dodecyl sulfate-polyacrylamide gels and transferred to Immobilion-PVDF membranes (Millipore, Billerica, MA, USA). Antigen-antibody complexes were detected using an electrochemiluminescence blotting analysis system (Millipore). The following primary antibodies were used: caspase-9 (cleaved Asp315; GeneTex, Irvine, CA, USA), caspase-3, cleaved-poly (ADP-ribose) polymerase (PARP), extracellular signal-regulated kinase (Erk) and p-Erk (Cell Signaling Technology, Danvers, MA, USA), Raf-1 and MALT1 (Santa Cruz Biotechnology, Dallas, TX, USA), and β-actin (Abcam, Cambridge, United Kingdom). The working dilution of primary antibodies was 1:1,000.

### Luciferase assay

CCA cells were plated in 60 mm tissue culture dishes at 70% confluence overnight to allow complete attachment. A human NF-κB promoter containing a luciferase reporter construct was then co-transfected with a pNL1.1.TK [Nluc/TK] vector containing NanoLuc luciferase (Promega, Madison, WI, USA) into CCA cells using the X-tremeGENE HP DNA Transfection Reagent (Roche). To monitor promoter activity after drug treatment, a Nano-Glo Dual-Luciferase Reporter assay (Promega) was performed. All reporter experiments were performed in duplicate and repeated three times.

### Microarray chips and data mining analysis

Total RNA extracted from cells with A260/280 and A260/230 ratios greater than 1.8 was incubated in chips using the Affymetrix Human U133 2.0 Plus platform, including RNA from regorafenib-treated and control KKU-100 and HuCCT1 cells. Raw intensities in CEL files were normalized, and potential upstream regulators were predicted by using GeneSpring software (Agilent Technologies, Santa Clara, CA, USA) with >2.0 fold-change as the cut-off. Furthermore, common signatures between high regorafenib doses versus controls and low regorafenib doses versus controls were determined in two cell line models. The *Z*-score and *P*-value of several candidate targets were then calculated by using the Ingenuity Pathway Analysis (IPA) online data tools for further experimental analysis and validation as described [40].

### Transient transfection

For transfection, HuCCT1 cells were plated at a confluence of 50–60% in 6 cm culture dishes. Different quantities of DNA (0.5, 1, or 3 μg) from the pCMV-SPORT6 backbone were then incubated with serum-free medium in a total volume of 500 μL, and then mixed with 500 μL of X-tremeGENE^™^ HP DNA transfection reagent (Roche) plus serum-free medium solution (ratio 1:3) to bring the final volume to 1 mL. This pre-mixture was then incubated at room temperature for 30 minutes and then added to HuCCT1 cells for 3 hours. The cells were further treated with RPMI medium overnight and analyzed after 72 hours.

### Patient demographics

We examined the demographic features of 100 patients with mass-forming CCA (MF-CCA) who underwent hepatectomies between 1989 and 2006 at the Department of Surgery, Chang Gung Memorial Hospital. The study was approved by the local Institutional Review Board of Chang Gung Memorial Hospital (clinical study numbers 99-2886B, 99-3810B, and 102-5813B). This study was conducted in accordance with the principles of the Declaration of Helsinki and with the relevant guidelines and regulations of Chang Gung Memorial Hospital. Written informed consent for immunohistochemical tumor analysis was obtained from each patient.

### MALT1 immunohistochemistry

MALT1 expression levels in the aforementioned 100 MF-CCA patients were examined by immunohistochemical staining. Tissue sections (4 μm) were prepared from formalin-fixed, paraffin-embedded hepatectomy specimens, and incubated with anti-MALT1 primary antibody (N2C2, 1:500 dilution; GeneTex) overnight at 4°C. After three 5 minute washes with TBST, bound antibody signal was visualized using Dako Labelled Streptavidin-Biotin2 (LSAB2) System-HRP (Dako A/S, No. K0675; Dako, Glostrup, Denmark). Control slides were incubated with secondary antibody only. For the assessment of immunohistochemical staining, the percentage of stained target cells was evaluated in 10 random microscopic fields of view per tissue section (400× magnification), and their average scores were subsequently calculated. Staining intensities were scored as 1 (mild), 2 (moderate), or 3 (strong). H scores were calculated as the percentage of positive staining (0–100) × the corresponding staining intensity (0–3). Specimens with H-scores of <120 or ≥120 were classified as having low or high expression, respectively (range: 50 to 300; median 120).

### Follow-up study

The follow-up evaluation included physical examinations and blood chemistry tests during each visit. Additionally, serum levels of CEA and CA 19–9 were measured, and the remnant liver was examined by ultrasound (US) every 3 months. When a new lesion was detected by US or elevated levels of CEA/CA 19–9 were noted, abdominal CT or magnetic resonance cholangiopancreatography (MRCP) was performed for confirmation. When patients complained of bone pain, bone scans were performed to detect metastasis. If any of the above mentioned procedures indicated recurrence, the patient was readmitted for a more comprehensive assessment, including angiographic evaluation or magnetic resonance imaging (MRI). The methods for treating recurrence included surgery, systemic chemotherapy, external beam radiotherapy, intraluminal radiotherapy, interventional radiological therapy, and conservative treatment.

### Xenograft animal model

Animal studies were performed with the approval of the Academia Sinica Institutional Animal Care and Utilization Committee (IACUC) or the Experimental Animal Ethics Committee of Chang Gung Memorial Hospital. In addition, all animal studies followed the US National Institute of Health (NIH) Guidelines for the Care and Use of Laboratory Animal protocols (Publication No. 85–23, revised 1996). Age-matched severe combined immune deficiency gamma (JAX™ NOD.Cg-Prkdcscid Il2rgtm1Wjl/SzJ; NOD-SCIDγ) male mice at 6 weeks old were used (Jackson Laboratory, Bar Harbor, ME, USA). For estimation of *in vivo* tumorigenicity, 5 × 10^6^ SNU-308 CCA cells were re-suspended in 100 μL of PBS and injected subcutaneously under the dorsal skin of mice. When the size of subcutaneous tumor grew to 0.5 cm, different treatments were initiated: the sham group received PBS treatment and the treatment group received regorafenib at a dose of 30 mg/kg by oral gavage five times per week. Tumor growth was monitored once a week by Vernier caliper measurement of two perpendicular tumor diameters (L and W). Tumor volume was calculated using the formula LW^2^/2. Body weights were measured weekly. Animals in the regorafenib group stopped receiving treatment when body weight decreased to below 80% of starting body weight. Tumor masses were harvested after 6 weeks.

### Rat orthotopic tumor graft

Eighteen adult male Sprague-Dawley (SD) rats (310 ± 14 g) were used in these experiments. Animals were divided equally (*n =* 6) into the following three groups: control (Group 1), gemcitabine/oxaliplatin treatment (Group 2), and regorafenib treatment (Group 3). The rats were housed in an animal room with a 12:12 h light-dark cycle (lights on from 08:00 AM to 08:00 PM) at an ambient temperature of 22°C. Food and water were provided *ad libitum*. The rats were administered 300 mg/L thioacetamide (TAA) via drinking water daily for up to 20 weeks^.^ The gemcitabine/oxaliplatin treatment group received gemcitabine (50 mg/kg, i.p.) and oxaliplatin (2 mg/kg, i.p.) once every 2 weeks over a 4 week period starting at the 21st week. The regorafenib treatment group received regorafenib (30 mg/kg, p.o.) every day for 5 days per week starting at the 21st week. The control group rats received i.p. injections of PBS according to the same schedule.

### Evaluation of treatment efficacy in rats by positron emission tomography

To evaluate the changes in glycolysis in live animals with liver tumors, we conducted 2-deoxy-2-[F-18] fluoro-d-glucose (FDG)-positron emission tomography (PET) studies in rats at the Molecular Imaging Center of Chang Gung Memorial Hospital. In total, 18 rats were treated with TAA and subjected to serial PET scanning on weeks 21, 23, and 25 using the Inveon™ system (Siemens Medical Solutions USA Inc., Knoxville, TN, USA). Equal numbers of animals were assigned to the control and treatment groups based on their baseline PET results. This ensured that the control and treatment groups possessed similar PET-positive rates. The details of radioligand preparation, scanning protocols, and determination of optimal scanning time have been described previously by our group [41]. Briefly, animals were fasted overnight prior to scanning. At 90 min post-^18^F-FDG injection (i.v.), 30 min static scans were obtained for all of the animals. All imaging studies were performed by using a temperature- (set to 37°C) and anesthesia- (2% isoflurane vaporized in 100% oxygen) controlled imaging bed (Minerve System, Esternay, France). PET images were reconstructed using the 2D ordered subset expectation–maximization method (4 iterations and 16 subsets) without attenuation and scatter corrections. All imaging data were processed using the PMOD image analysis workstation (PMOD Technologies Ltd., Zurich, Switzerland). The largest liver tumor for each animal was identified by careful investigation of all 3 image sets for each rat. ^18^F-FDG uptake into the largest liver tumor, as well as apparent normal liver tissue was quantified by calculating the standardized uptake value (SUV). These values were calculated according to the recommendations of the European Organization for Research and Treatment of Cancer [41]. The tumor regions of interest (ROIs) were determined by using transverse images of the selected tumors and measuring the largest diameter. The normal liver ROIs were also determined by using the same transverse images. The mean SUV (SUV_mean_) of the normal liver and tumor tissue was determined, and the tumor-to-liver radioactivity ratio was calculated for comparison.

### Statistical analysis

All data were presented as the mean ± SD. Differences between the experimental and control groups were calculated by using the Student’s *t*-test. Progression-free survival (PFS) and overall survival (OS) rates were evaluated with the Kaplan–Meier method. Several clinicopathological variables were considered for the initial univariate analysis, which was performed by using Log-rank test. The Cox proportional hazards model was applied for multivariate regression. SPSS for Windows (Version 17.0, Chicago, IL, USA) was used for statistical analysis. A value of *P* ≤ 0.05 derived from 2-tailed tests was considered statistically significant.

## SUPPLEMENTARY MATERIALS FIGURES AND TABLES


