# Supplementary material for: Identification of MALT1 as both a prognostic factor and a potential therapeutic target of regorafenib in cholangiocarcinoma patients
Source: Oncotarget. 2017 Dec 8;8(69):113444–59. doi: 10.18632/oncotarget.23049 (PMC5768338; doi:10.18632/oncotarget.23049)
Supplement: Supplementary file 1 [file oncotarget-08-113444-s001.pdf]

Identification of MALT1 as both a prognostic factor and a potential therapeutic target of regorafenib in cholangiocarcinoma patients

SUPPLEMENTARY MATERIALS

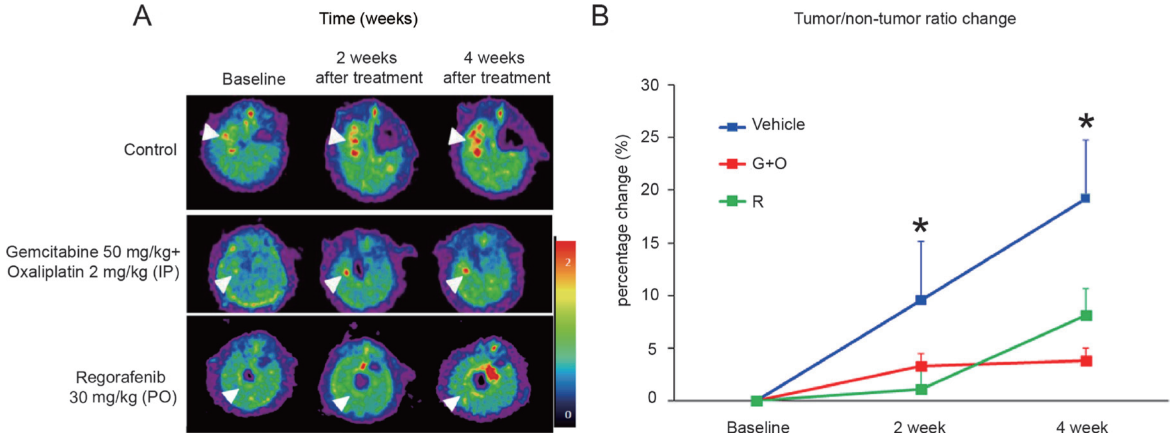

Supplementary Figure 1: Potent cell growth inhibition induced by regorafenib in the thioacetamide induced CCA rat animal model. **(A)** Left panel is representative PET scan coronal views of control and drug treated rats. **(B)** Right panel shows the change of the tumor-to-liver (T/L) ratio of the SUV in the control and drug treated groups. Gemcitabine/oxaliplatin and regorafenib treatment both induced significant decreases in the T/L ratio of the SUV after two to four weeks of treatment when compared with the control group ($P < 0.05$).

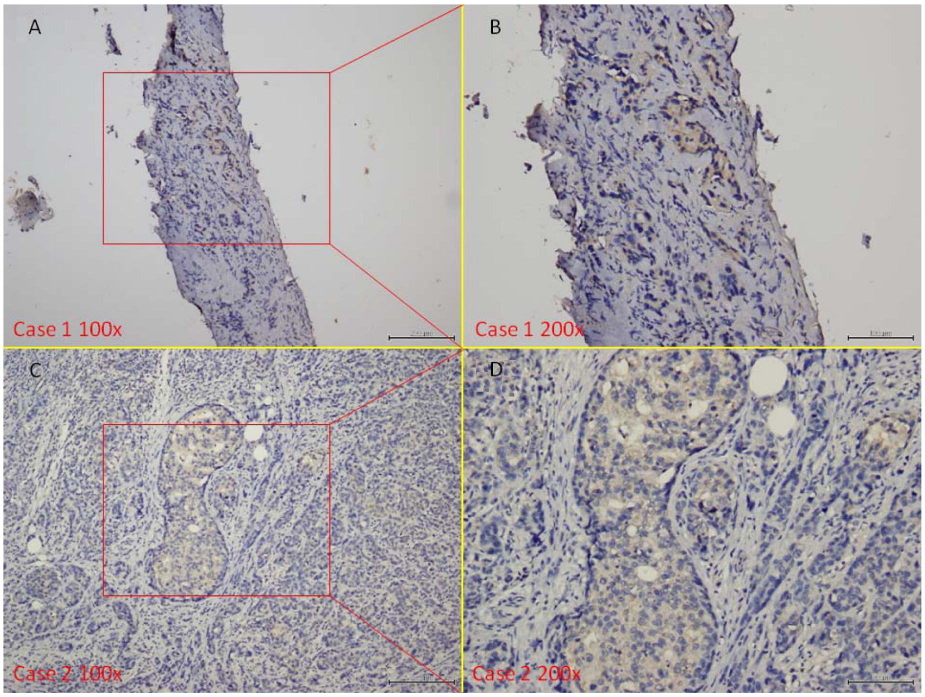

Supplementary Figure 2: High expression of MALT1 in human CCA samples. The immunohistochemical stain from two CCA patients who had responded to regorafenib. Scale bar = 50 μm. **(A–C)** In low-power field (×100), tumor and adjacent normal tissue were clearly differentiated in patients no.1 and no.2 respectively. **(B–D)** The immunohistochemical staining in high-power field (×400) showed overexpression of MALT1 in tumor tissue, but not in adjacent normal tissue in patients no.1 and no.2 respectively.

Supplementary Table 1: Analysis of perturbagen candidates by the gene signature of regorafenib treatment in SNU-1196, SNU-1079, and HuCCT1 cell lines in LINCS dataset

Perturbagen	Cholangiocarcinoma cell lines		
	SNU1196	SNU1079	HuCCT1
**ECH1**	98.5614	95.7328	90.0195
**MALT1**	95.4322	94.0608	92.9171
**ALAS1**	92.1505	92.7796	92.6832
**XRCC4**	99.4518	93.2487	N/D
**GPRC6A**	98.315	N/D	90.1925
**SPRED2**	98.244	94.9241	N/D
**PRDM1**	98.1332	97.7032	N/D
**HMGA1**	97.1264	91.6378	N/D
**MCL1**	96.8019	94.0444	N/D
**IFNB1**	96.7716	97.6938	N/D
**ZNF32**	96.7618	96.2755	N/D
**RPS6KA3**	96.519	94.4262	N/D
**VN1R2**	95.9409	94.0869	N/D
**MAST4**	95.6138	92.5555	N/D
**TAAR1**	95.6017	95.1259	N/D
**F5**	95.4345	98.894	N/D
**EPN2**	94.9301	90.9729	N/D
**CSNK2A1**	94.6394	N/D	92.8016
**UBTF**	94.3715	93.2427	N/D
**SYK**	94.223	92.9373	N/D
**LCMT1**	94.054	97.2416	N/D
**PIK3C2A**	93.8471	N/D	92.498
**OTUD3**	93.2226	94.4802	N/D
**PNRC1**	93.1605	92.0213	N/D
**CSNK2A2**	93.0726	94.5291	N/D
**GBA**	92.7374	N/D	91.2808
**DBI**	92.6762	91.9126	N/D
**ASH2L**	92.4213	92.274	N/D
**GALT**	92.1918	N/D	94.0245
**ZNF619**	91.884	94.6265	N/D
**TAF15**	91.7787	90.5861	N/D
**PTCH1**	91.4633	N/D	92.3513
**MPL**	91.1272	90.0528	N/D
**MRGPRD**	90.7831	90.4612	N/D
**MPHOSPH9**	90.6818	90.3652	N/D
**NAT10**	90.5876	92.1142	N/D
**MELK**	N/D	94.4698	90.2246
**PC**	N/D	92.6612	92.5273
**NFRKB**	N/D	92.4873	90.693
**ITGA4**	N/D	91.9163	95.7696
**COMT**	N/D	91.4661	96.9328
**GPX7**	N/D	90.8805	93.4624
**INSIG1**	N/D	90.7625	92.3135

The perturbagen candidates were queried from the regorafenib gene signature with 1.5 fold-change comparing with vehicle control in LINCS dataset. The perturbagen candidates were then prioritized by the connectivity score across the four cell lines of dataset, in which the pertubagen gene signature connected most strongly to that of regorafenib treatment. The connectivity score were cut off at 90. N/D, not determined.

Supplementary Table 2: Predicted transcription factor binding sites of *MALT1* by PROMO 3.0

YY1	HNF-1A	GR-alpha
GR-beta	STAT4	AP-1
c-Jun	FOXP3	XBP-1
NF-1	c-Ets-1	C/EBPalpha
STAT1beta	TFIID	RXR-alpha
P53	AP-2alphaA	Elk-1
TFII-I	Pax-5	Sp1
GCF	ER-alpha	TCF-4
GR	C/EBPbeta	

Supplementary Table 3: Clinicopathological features and MALT1 expression in patients with cholangiocarcinoma

	MALT1 low expression (n = 46)	MALT1 high expression (n = 54)	p
Age (years)	59.80 ± 12.95	60.22 ± 11.19	
Gender			0.642
Male	20 (43.5%)	21 (38.9%)	
Female	26 (56.5%)	33 (61.1%)	
Symptom			**0.026***
No	12 (26.1%)	5 (09.3%)	
Yes	34 (73.9%)	49 (90.7%)	
AST (IU/L)			0.205
≤34	28 (60.9%)	25 (48.1%)	
>34	18 (39.1%)	27 (51.9%)	
ALT (U/L)			0.830
≤36	27 (61.4%)	29 (59.2%)	
>36	17 (38.6%)	20 (40.8%)	
ALP (U/L)			**0.027***
≤94	22 (51.2%)	14 (28.6%)	
>94	21 (48.8%)	35 (71.4%)	
Bilirubin (total) (mg/dl)			0.103
≤1.3	42 (91.3%)	43 (79.6%)	
>1.3	4 (08.7%)	11 (20.4%)	
Albumin (g/dl)			0.059
≤3.5	7 (16.7%)	17 (34.0%)	
>3.5	35 (83.3%)	33 (66.0%)	
Serum CEA (ng/mL)			0.166
≤5	20 (58.8%)	18 (42.9%)	
>5	14 (41.2%)	24 (57.1%)	
Size (cm)			0.211
≤5	23 (51.1%)	20 (38.5%)	
>5	22 (48.9%)	32 (61.5%)	
Lymph node			0.071
Negative	35 (76.1%)	30 (58.8%)	
Positive	11 (23.9%)	21 (41.2%)	
Differentiated			0.967
Well	1 (02.2%)	2 (03.7%)	
Moderate	23 (50.0%)	28 (51.9%)	
Poorly	21 (45.7%)	23 (42.6%)	
Other	1 (02.2%)	1 (01.9%)	
Margin			**0.001***
Negative	41 (89.1%)	32 (59.3%)	
Positive	5 (10.9%)	22 (40.7%)	
Post Chemotherapy			0.828
Without (n = 48)	24 (52.2%)	27 (50.0%)	
With (n = 48)	22 (47.8%)	27 (50.0%)	
Post Radiotherapy			0.348
Without (n = 84)	42 (47.7%)	46 (52.3%)	
With (n = 12)	4 (08.7%)	8 (14.8%)	

*statistically significant by multi-logistic regression analysis: p = 0.006 for symptom.

Supplementary Table 4: Univariate analysis of factors influencing the overall survival of patients with MF-CCA

Factors	Survival Time (months)				*p*
	Median	95% CI of Median	3-year (%)	5-year (%)	
Gender					0.473
Male (n = 41)	19.04	12.31–25.76	27.2	24.2	
Female (n = 59)	14.14	10.86–17.42	25.5	14.2	
Age					0.322
≤60 (n = 49)	17.16	10.44–23.88	29.5	24.4	
>60 (n = 51)	14.53	11.24–17.82	23.3	12.1	
Symptoms					**0.001**
Negative (n = 16)	46.26	NA	61.6	46.9	
Positive (n = 80)	12.99	9.76–16.21	19.2	12.5	
AST (IU/L)					0.147
≤34 (n = 53)	15.81	7.17–24.46	32.3	24.9	
>34 (n = 45)	15.85	5.60–26.09	20.9	11.9	
ALT (IU/L)					0.283
≤36 (n = 56)	15.81	9.05–22.58	30.0	20.2	
>36 (n = 37)	15.85	8.52–23.17	22.1	10.1	
ALP (IU/L)					**0.002**
≤94 (n = 36)	26.86	14.77–38.95	40.3	27.5	
>94 (n = 56)	10.72	6.30–15.14	16.1	10.0	
Bilirubin (total) (mg/dL)					0.539
≤1.3 (n = 85)	15.85	9.41–22.28	27.8	18.4	
>1.3 (n = 15)	14.53	6.40–22.67	17.8	17.8	
Albumin (g/dL)					**0.006**
≤3.5 (n = 24)	5.00	3.62–6.38	16.7	12.5	
>3.5 (n = 68)	19.99	14.94–25.04	28.0	17.7	
Serum CEA (ng/dL)					0.064
≤5 (n = 38)	20.88	9.70–32.05	38.6	23.4	
>5 (n = 38)	12.72	8.47–16.97	13.6	13.6	
Margin					**<0.001**
Negative (n = 73)	19.99	14.49–25.49	35.5	24.7	
Positive (n = 27)	4.70	2.61–6.79	0	0	
Size					**0.013**
≤5 cm (n = 43)	20.88	12.38–29.38	39.4	30.3	
>5 cm (n = 54)	13.18	8.88–17.49	17.2	8.6	
Lymph node					**0.023**
Negative (n = 65)	23.01	15.32–30.71	33.4	20.6	
Positive (n = 32)	12.72	8.99–16.46	15.0	15.0	
Histological differentiation					0.949
Well (n = 3)	6.08	0.72–11.45	33.3	33.3	
Moderate (n = 51)	19.04	12.46–25.61	28.5	20.7	
Poor (n = 44)	14.40	10.88–17.92	24.8	15.8	
Others (n = 2)	10.72	NA	0	0	
MALT1 expression					**<0.001**
Low (n = 46)	38.60	20.16–57.04	53.5	37.3	
High (n = 54)	6.08	3.82–8.34	0	0	
Post-op Chemotherapy					0.322
Without (n = 51)	14.40	0.00–30.58	35.6	26.3	
With (n = 49)	15.81	11.26–20.37	17.6	11.0	
Post-op Radiotherapy					0.056
Without (n = 88)	14.70	8.80–20.59	30.2	21.0	
With (n = 12)	7.17	0.00–27.65	0	0	

CI: confidence interval; AST: aspartate aminotransferase; ALT: alanine aminotransferase; ALP: alkaline phosphatase; CEA: carcinoembryonic antigen; CA 19-9: carbohydrate antigen 19-9; IU: international unit; op: operation.

Supplementary Table 5: Multivariate analysis of factors influencing the overall survival of patients with MF-CCA

Factors	Relative Risk (95% confidence interval)	*P*-value
Symptoms (positive/negative)	2.08 (0.83–5.23)	0.117
ALP (>94 g/dL/≤94 g/dl)	0.91 (0.48–1.74)	0.780
Albumin (>3.5 g/dl/≤3.5 g/dl)	0.64 (0.34–1.20)	0.163
Margin (positive/negative)	2.35 (1.28–4.33)	**0.006**
Tumor size (>5 cm/≤5 cm)	1.21 (0.69–2.12)	0.499
Lymph node (positive/negative)	1.01 (0.57–1.79)	0.962
MALT1 expression (high/low)	13.63 (6.19–29.99)	**<0.001**

ALP: alkaline phosphatase; CEA: carcinoembryonic antigen.

Supplementary Table 6: The primer sets of qPCR

ALAS1_F	5′ CCTTggCCTTAgCAgTTTTg 3′
ALAS1_R	5′ CCAAgATgATggAAgTTggg 3′
ECH1_F	5′ TgATgTCACggAggTACCAg 3′
ECH1_R	5′ TggTgCAggAAAAATgTTCA 3′
MALT1_F	5′ TTgTTCTTCCgCCCCTgC 3′
MALT1_R	5′ TgACCTTCAgCCCCTAC 3′
IL-1a_F	5′ CCgTgAgTTTCCCAgAAgAA3′
IL-1a_R	5′ ACTgCCCAAgATgAAgACCA3′
IL-8_F	5′ CAAgAgCCAggAAgAAACCA3′
IL-8_R	5′ AgCACTCCTTggCAAAACTg3′
GAPDH_F	5′gCCAAggTCATCCATgACAACT 3′
GAPDH_R	5′gAggggCCATCCACAgTCTT3′
